# Modeling the interplay between emotion regulation, self-efficacy, and L2 grit in higher education

**DOI:** 10.3389/fpsyg.2022.1013370

**Published:** 2022-09-22

**Authors:** Shengtao Zheng, Tahereh Heydarnejad, Amhara Aberash

**Affiliations:** ^1^School of Foreign Studies, Minnan Normal University, Zhangzhou, China; ^2^Department of English Language, Faculty of Literature and Humanities, University of Gonabad, Gonabad, Iran; ^3^Department of English Language, Jimma University, Jimma, Ethiopia

**Keywords:** higher education, EFL university professors, ER, self-efficacy, L2 grit

## Abstract

Teaching in higher education is critical and fraught with potential vicissitudes, which necessitates the presence of efficient professors armed with positive attributes to perform effectively. Although it is generally accepted that emotion regulation (ER) has numerous benefits for language teachers, in particular university professors, little is known about how it interacts with two other important constructs, i.e., self-efficacy and L2 grit. Furthermore, the effect of ER on L2 teacher grit has not been sufficiently investigated. To fill this gap, the current study was to test a structural model of English as a Foreign Language (EFL) university professors’ ER, self-efficacy, and L2 grit. The participants were 356 Iranian EFL university professors who completed the Language Teacher Emotion Regulation Inventory (LTERI), the Teacher Sense of Efficacy Scale (TSES), and the L2-Teacher Grit Scale (L2TGS). The results of Structural Equation Modeling (SEM) revealed that ER and self-efficacy were strong predictors of L2 grit. Moreover, the significant role of self-efficacy on ER was discovered. The implications of this study may foster effective teaching in higher education, particularly during the COVID-19 pandemic and its impacts on education.

## Introduction

Teaching at university is very demanding, and university professors should use a wide range of skills to teach and act effectively. It is necessary that teachers, in particular university professors be aware of what qualities and skills enhance an efficient instruction. Considering the indisputable relevance of teacher emotion and cognition to their effectiveness, it is prominent that teachers utilize useful methods in order to manage experienced emotions and boost cognitive affairs. In the domain of effective teaching, in particular language teaching, it seems plausible to presume that a higher level of emotional competencies helps teachers to manage and modify their performance. As Wubbels and Levy (1991) put it, emotional competencies affect not only the effectiveness of instructors but also the cognitive and emotional development of students, leading to successful and effective teaching. ER as a complex process refers to different strategies used for initiating, hampering, or modifying individuals’ position or behavior in a specific circumstance (Gross, 1998a). Teacher emotion regulation (TER) refers to “their capability to manage emotional experiences and expressions” (Burić et al., 2017, p. 2). Through the lens of emotion regulation (ER), teachers are able to evaluate and modify the intensity and duration of the emotional experiences at the workplace (Chang and Taxer, 2020). In ever-changing and challenging teaching contexts, TER acts as a shield to protect and immune teachers in the face of plights. The role of TER is more significant in language teaching because it is an emotionally charged endeavor (Tsang and Jiang, 2018; Richards, 2020).

Another key construct on the road to university professors’ professional growth is self-efficacy. According to Bandura (1997, 1986), self-efficacy may be described as an individual’s perception of their own capacity to successfully complete or display an activity or sequence of behaviors in a certain setting. From another view point, self-efficacy is defined as a cognitive, motivational, emotional, and selection-based regulator of performance (Bandura, 1994). Self-efficacious people set more challenging goals and envisage success. Based on Bandura (1997), self-efficacy beliefs play a significant role in the regulation of motivation and boost individuals’ confidence in their abilities, which may also decrease the stress and depression that self-efficacious people experience in threatening or demanding conditions.

More specifically, self-efficacy beliefs influence the kind of activities and environments that individuals select. Self-efficacy beliefs affect individuals’ thinking, their future actions, their coping strategies while facing emotional demands, and the attempts they put forth in a given endeavor (Bong and Clark, 1999; Bandura, 2012). Self-efficacy is both a personal and social construct because each person functions individually and collectively. Individuals’ worries about the effectiveness of the group as a whole have an impact on the activities they choose to pursue together, how much attempt they make to perform it, their persistence and tolerance, and their probability of success (Bandura, 1994). Collective efficacy refers to a group’s common beliefs in its ability to achieve goals and desired tasks (Schunk and Pajares, 2002). Teacher self-efficacy is defined as to the extend they have conviction to successfully execute behaviors to achieve educational objectives (Gibson and Dembo, 1984). Efficacious teachers tend to support, persevere during challenges, open to new ideas, and implement helpful teaching strategies (Gordon et al., 2022; Ma, 2022). Teachers with higher perceived self-efficacy are more engaged in their work activities (Burić and Macuka, 2017; Li et al., 2019) and willing to implement curriculum reform (Cerit, 2019). Efficacious teachers also deal with Students’ misbehavior and demotivation more efficiently (Burić and Kim, 2020).

The metaphor of teacher L2 grit is a personality trait resulting from an amalgamation of perseverance of attempt and teaching passion for long-term objectives (Sudina et al., 2021). Teacher L2 grit is quite uncharted territory, which awaits further research, and its relationships with other teacher-related construct is still under a shadow, in particular in higher education. Teacher L2 grit might be of immediate relevance to TER and self-efficacy. Despite its dominant role, and perhaps because of the novelty of teacher L2 grit, research has not focused on the relationship between TER, self-efficacy views, and L2 grit. Especially within the realm of higher education. To this end, the present study set out to model the relationships between ER, efficacy beliefs, and L2 grit among EFL university professors. Exploring the relationship between these constructs, which are conductive to effectiveness may envision a picture of EFL of university professors’ ER, self-efficacy, and L2 grit and accordingly their effectiveness.

## Literature review

In the following sections, the relevant literature on teacher emotion regulation, teacher self-efficacy, and teacher L2 grit is briefly reviewed.

### Teacher emotion regulation

ER is “a heterogeneous set of physiological, behavioral, and cognitive processes” (Gross and John, 2003, p. 348) that individuals apply to manage their emotional experiences. As Gross (1998a) stipulated, emotions are processes that unfold over time, and ER is a dynamic process that extends beyond a single episode. That is, a specific situation is attended to, appraised, adjusted, and generates emotional responses (Gross, 2014). According to Gross and Barrett (2011), the activation of a regulatory objective, the engagement of regulatory mechanisms, and the alteration of the emotion trajectory are the three components that combine to produce ER. The activation of a goal is the first constituent of ER (Gross and Barrett, 2011). The activation of a goal might take place either inside oneself (via intrinsic ER) or within another individual (extrinsic ER) (Gross and Barrett, 2011). Intrinsic ER describes instances in which people modulate their own emotions (ER in self), while extrinsic ER is when one person regulates another person’s emotions (ER in another). Specialists consider intrinsic ER for studies relevant to adults (Gross, 1998b), while in working with infants and children, extrinsic ER is highlighted (Cole et al., 2004). On some occasions, both intrinsic and extrinsic ER are applied; for instance, in one situation, a person may regulate another person’s emotions (extrinsic regulation) to calm himself/herself down (intrinsic regulation) (Gross, 2014).

The second constituent of ER refers to the engagement of the processes involved in changing emotion trajectory. Different processes are involved in ER that may be explicit or implicit (Gross, 2014). If ER happens with conscious awareness, it is considered as explicit ER. For instance, when a person tries to consider the bright side of a bad happening to cheer themselves up, they are employing explicit ER (Gross, 2014). ER activities may also happen unconsciously and implicitly (Gross, 2014). For example, when individuals quickly turn their attention away from potentially annoying materials (Gross, 2014). In previous studies, explicit and implicit processes in ER are considered separately (Masters, 1991). However, it is recommended to consider ER processes as a spectrum that extends from overt, intentional, and deliberate control to covert, unconscious, seamless, and automatic regulation (Gyurak et al., 2011; Vadivel and Beena, 2019). The effect of ER on emotion dynamics is the third core feature of ER (Thomas, 1990). In this regard, Gross (1998b) pointed out that the ER can have an increased or decreased latency, rising time, size, length, or offset depending on the individual’s objectives (Gross, 1998b). Moreover, as the emotion develops, ER may alter the degree to which the various components of the emotional reaction cohere (Dan-Glauser and Gross, 2013; Vadivel et al., 2021).

Over the years, different ER models were developed to describe the involved procedures. The Hot/Cool System of ER, for example stimulates the processes involved in ER into willpower (Mischel and Ayduk, 2004). It is imagined that the cool system generated in adulthood helps individuals to keep calm in intensive emotional disturbances. Hot system developed in childhood working as quick emotional processing (Sutton and Harper, 2009; Liu et al., 2021). Another suggested model for ER is the Resources or Strength Model, which is supported by self-regulation theory (Schmeichel and Baumeister, 2004). A more comprehensive model is the process model of ER (Gross, 1998a,b, 2014; Gross and Thompson, 2007), which stipulated five temporal points in the process of emotion generation as follows: situation choice, situation adjustment, attentional deployment, cognitive transformation, and reaction modulation. According to the definitions provided by (Gross, 1998a,b), these five points illustrate five different families of ER processes.

The first four families of strategies (i.e., situation choice, situation adjustment, attentional deployment, cognitive transformation) are grouped as antecedent-focused. But, the fifth set (i.e., reaction modulation) modulates the aspects of the fully developed emotional response (Gross and Thompson, 2007). Situation selection, as the first step in ER, refers to employed strategies to decrease the likelihood of any happening that may trigger an undesirable emotion. Situation modification processes enhance changing the features of an occasion that evoke a specific emotion. Attentional deployment refers to individuals’ attempts to redirect their attention to regulate their emotion. Cognitive change mainly alters the cognitive appraisal of a situation that triggers emotional experience by reforming an individual’ thinking either by changing the situation or an individual’ capacity to modify it. Response modulation, as the last process, refers to various strategies to intensify, reduce, or extend the physiological, experiential, or behavioral responding components of emotional responses (Gross, 2014).

Regarding Language TER, recently a model was proposed by Heydarnejad et al. (2021b). This model was generated based on the existing literature on TER (e.g., Burić et al., 2017; Chang, 2020; Chang and Taxer, 2020; Richards, 2020; Alipour et al., 2021; Chen and Cheng, 2021), ER (Gross and Thompson, 2007; Taxer and Gross, 2018) in particular, Gross’ process model of ER (1998a,b, 2014). This suggested model for language TER involves six dimensions as following: situation selection, situation modification, attention deployment, reappraisal, suppression, and seeking social support. Situation selection, situation modification, and attention deployment were formulated based on Gross’ process model of ER (1998a,b, 2014). The two dimensions of reappraisal and suppression were generated based on Gross and John’s findings (2003). Seeking social support, as the last dimension originated from Jennings and Greenberg (2009) as well as Taxer and Gross (2018).

As reviewing the existing literature on TER, in particular university professors reflected, this important concept is still in its infancy and calls for more research to fill this gap. The existing literature confirmed the contributions of ER to other teacher related constructs. For example, Morris and King (2018) investigated the influence of ER in controlling the frustration experienced by university professors in their classes. They found that using contextually dependent ER behaviors assist language university professors in improving their confidence levels and manage their stress. In another study by Chang and Taxer (2020), TER strategies regarding classroom misbehavior were examined. According to their outcomes, those teachers who reappraised were less touched by their learners’ misbehavior; they also experienced less suppression. Taking a similar path, Morris and King (2018) found that emotion regulatory strategies were the best mechanism for managing frustrations among EFL university professors. In a mixed method study, the learners’ attitudes toward their teachers’ emotions and ER in the teaching processes were investigated by Jiang et al. (2016). Based on their findings, antecedent-focused emotion regulatory strategies were preferable to response-focused ones. Furthermore, they concluded that teachers’ reappraisals increased positive-emotion expressions. Recently, Fathi et al. (2021) investigated the relationship between teacher reflection, self-efficacy, burnout, and ER. Their findings suggested that teacher self-efficacy and reflection predicted ER. The negative relationship between ER and burnout was also confirmed by this study. Parallel with this line of inquiry, the mediator roles of teacher self-efficacy and ER on psychological wellbeing in an EFL context was concluded (Xiyun et al., 2022).

### Teacher self-efficacy

Self-efficacy refers to individuals’ impressions about their abilities to execute behaviors, leading to specific achievements (Bandura, 1982). Self-efficacy does not necessarily involve affective reactions toward the self, but it is mainly a cognitive judgment of one’s ability that attach diverse weights to different sources of information when arriving at such perception (Bong and Clark, 1999). Teacher self-efficacy is defined as “the teacher’s conviction in his or her capacity to plan and carry out a course of action necessary to effectively complete a given task in a specific situation” (Tschannen-Moran et al., 1998, p. 22). Self-efficacy enhances teachers inter and intra-relationships (Martin and Mulvihill, 2019) and increases their passion for instructional practices (Moè, 2016). Furthermore, teachers’ efficacy beliefs influence Students’ motivation, achievement, and efficacy (Tschannen-Moran et al., 1998). From another perspective, it is evident that teachers’ self-efficacy predicts their attitude, teaching style, self-regulation, commitment, motivation, and effectiveness (Barni et al., 2019; Fathi and Saeedian, 2020; Fathi et al., 2021; Heydarnejad et al., 2021a; Amirian et al., 2022) and supports Students’ academic achievement (Martin and Mulvihill, 2019).

In the current study, the Teachers’ Sense of Efficacy Scale was utilized (Tschannen-Moran and Hoy, 2001), which includes three subscales (efficacy in student engagement, efficacy in instructional strategies, and efficacy in classroom management). The efficacy in student engagement focuses on the teachers’ efficacy beliefs, which in turn fosters support for Students’ learning and motivation. The instructional strategies consider the instructor’ s capability to modify teaching to achieve learner needs. The classroom management evaluates the instructor’s efficacy in managing learner behavior (Tschannen-Moran and Hoy, 2001; Azari Noughabi et al., 2022; Shirvan and Alamer, 2022).

Teacher self-efficacy is generated from Bandura’s self-efficacy theory, which concentrates on the teacher’s beliefs of their abilities to involve their students in the learning processes effectively with the aim of realizing teaching and learning objectives efficiently (Tschannen-Moran et al., 1998; Sudina et al., 2020; Heydarnejad et al., 2021a). Bandura (1997) stipulated sources of efficacy beliefs as mastery experience, vicarious experience, social or verbal persuasion, and physiological or affective states. Mastery experience has the most influential role in self-efficacy beliefs, suggesting that successful performance increases self-efficacy (Bandura, 1997; Zarrinabadi et al., 2022). In other words, the perception of successful performance facilitates perceived self-efficacy and ensures future proficiency and success. In contrast, the perception of unsuccessful performance weakens efficacy beliefs and leads to the expectation that future performance will also be inefficient.

As Helsin (1997) stated, self-mastery may be accomplished by disassembling a difficult challenge into its component parts, which will increase the likelihood of one’s first achievement. The second significant influence is rooted in observing other similar people to perform a behavior successfully. It can provide individuals with ideas about successful performance (Tompson and Dass, 2000). The third source is social or verbal persuasion which is originated from other people. Successful persuasion enhances individuals’ beliefs in their abilities and ensures that future achievement is achievable (Wolfe, 1997). Whereas, negative persuasion may decrease self-beliefs. According to Schunk and Pajares (2002), the most contributing influence of social persuasion revolves around initiating a task, trying new strategies, and attempting hard to succeed. The fourth source, psychological and affective states such as engagement, anger, and anxiety provide information about efficacy perception and enhance the sense of proficiency. Therefore, attempting to reduce negative experiences and modifying negative debilitative states to positive facilitator states may help amend the perceived self-efficacy beliefs. The influence of these sources on self-efficacy is not automatic, but cognitively weighted and assessed (Bandura, 1997).

The importance of teacher self-efficacy among psychologists, educationalists, and social scientists has fueled intensive research over the last decades. For instance, Burić and Kim (2020) found that teacher self-efficacy predicts classroom management, cognitive activation, and supportive climate. Similarly, Burić and Frenzel (2019) concluded that teacher self-efficacy is associated with anger in a negative direction. In the same vein, Li et al. (2019) affirmed that teachers’ work engagement and self-efficacy were positively related. The beneficial effect of teachers’ motivations on their self-efficacy, openness to change, and self-transcendence was confirmed by Barni et al. (2019). To picture the possible impact of teachers’ self-efficacy and collective teacher efficacy on their psychological wellbeing, a study was conducted by Fathi et al. (2020) in the Iranian EFL context. Their data analysis indicated that teacher self-efficacy was a stronger predictor of psychological wellbeing than collective teacher efficacy. In a recent study, the contribution of critical thinking and self-efficacy beliefs to teaching style preferences among university professors was concluded (Amirian et al., 2022). In the same vein, the contributions of EFL teachers’ self-efficacy and creativity on their Students’ academic achievement was confirmed by Ma (2022).

### Teacher L2 grit

The Grit theory was introduced by Duckworth (2016), which emphasized that the reciprocal relationships of enthusiasm and persistence affected individuals’ potential to achieve their goals effectively. As Duckworth et al. (2007) defined grit refers to “working strenuously toward challenges, maintaining effort and interest over the years despite failure, adversity, and plateaus in progress” (pp. 1,087–1,088). Thus, enthusiasm and persistence are the key constructs in grit formation (Duckworth, 2016). Enthusiasm is a feeling of eager interest in or desire for a special subject or activity. Persistence is an element of the trait-level grit that provoke individuals to dedicate themselves to competence activities with long-term success (Duckworth, 2016). According to Dale et al. (2018), Grittier people have positive attitudes toward life and show high job dedication. Grit is enhanced when individuals understand the difference between high-priority and low-priority objectives and learn how to manage their energies (Hejazi and Sadoughi, 2022; Lan, 2022).

Teacher grit is defined as the perseverance of effort and consistency of interest (Duckworth et al., 2007; Pawlak et al., 2022). Teacher grit attributes teachers in handling their stress which leads to effective teaching (Alamer, 2021; Sudina et al., 2021) and work engagement (Maiers and Sandvold, 2017; Namaziandost and Çakmak, 2020). As it was evidenced, gritty teachers devote their energy to their teaching for a long time and enjoy their teaching procedures even if they encounter problems at the workplace (Sudina et al., 2021). Although studies in the realm of language learners’ grit were quite rosy in recent years (e.g., Khajavy et al., 2020; Wei et al., 2020; Cheng, 2021; Khajavy, 2021; Yang et al., 2022), language teachers’ L2 grit and its correlates are quite untouched (Sudina et al., 2021; Li, 2022). As Teimouri et al. (2020) discussed, this shortage can be attributed to a lack of domain-specific scales to measure grit in Second Language Acquisition (SLA). As it was concluded by Cormier et al. (2019) and Yang et al. (2022), grit is a domain-specific construct. This idea encouraged Sudina et al. (2021) to propose the model of L2 grit among language teachers. Teacher L2 grit is considered a personality trait that involves perseverance of effort and consistency of interest (Sudina et al., 2021). In this regard, Sudina et al. (2021) developed and validated L2-Teacher Grit Scale (L2TGS) to evaluate the L2 grit among language teachers.

Due to the recent introduction of language teachers’ L2 grit in 2021, the existing gaps in empirical studies echo a clear need to investigate the correlates of language teachers’ L2 with other teacher-related constructs. Recently, Ashkani et al. (2021) conducted a study to inspect teachers’ cognitive and behavioral manifestations of pedagogical beliefs and how teacher grit influences these two constructs in EFL contexts. Based on their findings, the grittier teachers presented strong associations between their beliefs and actual instructional practices. Moreover, they concluded that teacher grit predicted the relationships between EFL teachers’ self-reported pedagogical beliefs and their actual practices. Confirming the scant attention to L2 teacher grit, a theoretical analysis was completed by Xu (2022), in which the theoretical and empirical literature related to teachers’ hope, trust, and grit were reviewed. Shabani et al. (2022) examined to what extend pedagogical thoughts vary as a function of EFL teachers’ levels of grit. Their findings revealed that there were significant differences between low grit and high grit teachers considering the subscales of pedagogical thoughts. In the same line of inquiry, Liu (2022) concluded that gritty teachers are more motivated and enjoy the experience of language Instruction. In the realm of university students, Baierschmidt (2022) highlighted the significant role of grit as a predictor of foreign language proficiency.

### Objectives of the present study

In ever-changing and challenging teaching contexts, teachers in general and university professors, in particular, are exposed to various emotional experiences at the workplace. On such occasions, they need to be equipped with self-aid constructs to help them decide and act effectively. Through the lens of ER and self-efficacy, university teachers are expected to sustain their interest and make more effort to accomplish the established goals (L2 grit). Despite this, it appears that university TER and L2 grit are uncharted territories that await further research. Only within the last decades, TER has generated considerable attention from educators and researchers (Jiang et al., 2016; Taxer and Gross, 2018; Chang, 2020; Chen and Cheng, 2021), although it is virtually unexplored in the L2 domain (Richards, 2020; Alipour et al., 2021; Heydarnejad et al., 2021b). The same is true for L2 grit with emphasis that this concept was generated in 2021 and echoes urgent needs for further research.

**FIGURE 1:**
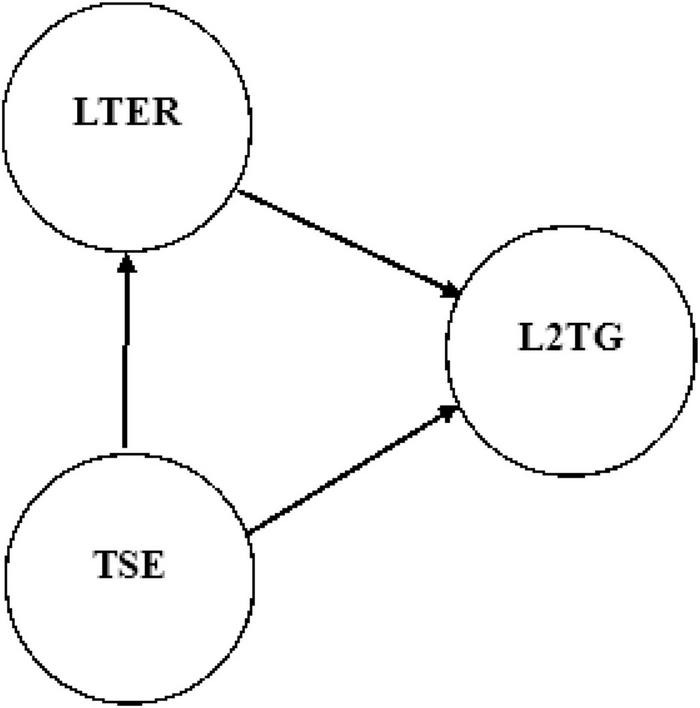
Theoretical structural equation model.

Moreover, TER and L2 grit as well as their influences on other factors and constructs conductive to effective teaching remained relatively unexplored in the field of second/foreign language education. Having attributed this gap, this study sought to propose a model to portray the relationships between ER, self-efficacy beliefs, and L2 grit with the prospect of shedding light on these issues and initiating further research (see Figure 1). The findings of this research may be both theoretically and practically significant. Such a study provides information to be taken into consideration by policymakers, language planners, curriculum designers, language teachers, university professors, as well as learners and their parents. Furthermore, the result of this study could provide Iranian EFL teachers and researchers with an awareness that can help them advance the more meaningful and effective teaching and learning strategies. To this end, the following research questions were posited:

RQ1: To what extent does EFL university professors’ ER predict their L2 grit?

RQ2: To what extent does EFL university professors’ self-efficacy predict their L2 grit?

The following null hypotheses were formulated based on the above-mentioned research questions:

H01. EFL university professors’ ER does not predict their L2 grit.

H02. EFL university professors’ self-efficacy does not predict their L2 grit.

## Materials and methods

In the following, the methodological steps that were taken in conducting this study were demonstrated:

### Participants

The study participants consisted of 356 Iranian EFL university professors teaching at different universities in Iran. The target population were chosen based on convenience or opportunity sampling procedures. To achieve generalizability, variation in years of teaching experience, age groups, genders, and universities/cities where university professors teach were considered during the data collection processes. Among 356 participants, there were 195 male and 161 females. Their age range between 29 and 52, and 1–28 years of teaching experience. All of the university professors were Ph.D. holders or Ph.D. candidates, and they majored in different branches of English: English Teaching (143), English Literature (93), English Translation (74), and also linguistics (46).

### Instruments

The following instruments were employed in the current research:

#### The language teacher emotion regulation inventory

To explore university professors’ ER strategies, LTERI was utilized. This instrument was developed and validated by Heydarnejad et al. (2021b), and the validity and reliability for all sub-scales of the LTERI were examined in two educational contexts (school and university), and the results of Cronbach’s alpha were acceptable (ranging from 0.718 to 0.814). To complete this instrument, the university professors are required to consider similar situations from their teaching experiences and choose their preferred ER strategies. This instrument involves 27 items on a five-point Likert scale anchored by 1 (“never”) and 5 (“always”) with six components, i.e., situation selection (e.g., I avoid conflicting or emotionally disturbing situations in the staff room.), situation modification (e.g., When an unpleasant discussion is raised in my classes, I try to change the topic.), attention deployment (e.g., If I feel frustrated in language classes, I try to engage myself in different class activities to forget it.), reappraisal (e.g., If for some reasons, I feel upset at work, I remind myself of my goals in my life.), suppression (e.g., If I feel helpless in my language classes, I disregard that.), and seeking social support (e.g., When I feel hopeless in my language classes, I seek advice from experts such as psychologists and school counselors.). In this study, the reliability of the LTERI estimated through Cronbach’s alpha was acceptable (ranging from 0.743 to 0.911).

#### The teacher sense of efficacy scale

The TSES (long form), developed and validated by Tschannen-Moran et al. (1998), was employed to gauge university professors’ self-efficacy beliefs. This instrument includes 24 items on a 9-point Likert scale with three subscales: (1) efficacy in student engagement (e.g., How much can you do to help your students think critically?), (2) efficacy in instructional strategies (e.g., How much can you do to foster student creativity?), and (3) efficacy in classroom management (e.g., How well can you implement alternative strategies in your classroom?). The reliability of the instrument was supported by the findings of Amirian et al. (2022). Based on the report of Cronbach’s alpha, the reliability of the TSES was acceptable (ranging from 0.756 to 0.891) in the current research.

#### The L2-teacher grit scale

The L2TGS, designed and validated by Sudina et al. (2021), was applied to inspect the university professors’ L2 grit. This instrument includes 14 items on a 5-point Likert scale with two subscales: perseverance in teaching (e.g., I am determined to withstand the work demands of the teaching profession) as well as passion and purpose in teaching (e.g., I manifest excitement in my teaching profession for a long time). This instrument is domain-specific and developed for evaluating EFL/ESL teachers’ grit. The report of Cronbach’s alpha for L2TGS was 0.944, which indicated acceptable reliability.

### Procedures

The data collection of this phase was started in February and ended in April, 2022 through a web-based platform. That is, the participants received an electronic survey form including the LTERI, the TSES, and the L2TGS through Google Forms. Conducting the electronic survey enables researchers to collect data from different regions with varying age groups and teaching experiences. The return rate was 89.2% and 356 forms were received. Each section in the electronic survey form was designed to be necessarily linked, thus no data were missed.

### Data analysis

The normality of the data was explored via Kolmogorov-Smirnov Test. As the data were normally distributed, CFA and SEM using LISREL 8.80 were conducted. SEM is a robust multivariate procedure used to take a confirmatory hypothesis-testing approach for the proposed structural theory (Schreiber et al., 2006). An SEM model involves two parts, the measurement model and the structural model (Kunnan, 1998). The measurement model is used to examine the relationships between the observed variables and latent variables. The structural model is used to gauge the relationships between the latent variables. Before testing a structural model, all the latent variables should be validated using CFA (Hair et al., 1998).

## Results

The results of the statistical analysis computed by the collected data are reported in this section. The descriptive statistics of EFL university professors’ ER, self-efficacy beliefs, and L2 grit are displayed in the following table.

Based on Table 1, among language TER strategies, reappraisal (*M* = 4.278, *SD* = 0.593) and attention deployment (*M* = 3.939, *SD* = 0.636) got the highest mean scores, whereas the mean score of suppression was the least (*M* = 3.503, *SD* = 0.767). Furthermore, among the components of self-efficacy beliefs, instructional strategies (*M* = 7.229, *SD* = 1.050) presented the highest mean scores. Efficacy in student engagement (*M* = 6.816, *SD* = 0.777) and efficacy in classroom management (*M* = 6.234, *SD* = 1.003) were the subsequent subscales of self-efficacy beliefs. Considering teacher L2 grit, the mean scores of subscales were as following: perseverance in teaching (*M* = 6.748, *SD* = 0.780) and Passion and Purpose in Teaching (*M* = 6.186, *SD* = 1.076), respectively. Then, to gauge the normality distributions of the data and consequently decide on employing a suitable statistical method for the current study, the Kolmogorov-Smirnov Test was utilized. In the following table, the result of the Kolmogorov-Smirnov Test is provided.

**TABLE 1:** Descriptive statistics.

Instrument	Subscales	N	Minimum	Maximum	Mean	SD
ER	Situation selection	356	1.00	5.00	3.627	0.722
	Situation modification	356	1.00	5.00	3.703	0.527
	Attention deployment	356	1.00	5.00	3.939	0.636
	Reappraisal	356	1.00	5.00	4.278	0.593
	Suppression	356	1.00	5.00	3.503	0.767
	Seeking social support	356	1.00	5.00	3.546	0.606
Self-efficacy	Efficacy in student engagement	356	1.00	9.00	6.816	0.777
	Efficacy in instructional strategies	356	1.00	9.00	7.229	1.050
	Efficacy in classroom management	356	1.00	8.38	6.234	1.003
L2 grit	Perseverance in teaching	356	1.00	9.00	6.748	0.780
	Passion and purpose in teaching	356	1.00	8.83	6.186	1.076

As Table 2 displays, the data were normally distributed because the sig value for all the instruments and their subscales were higher than 0.05. Therefore, parametric methods could be used to examine the related research hypotheses. In this regard, the LISREL 8.80 statistical package was utilized to inspect the structural relations between ER, self-efficacy, and L2 grit. The chi-square magnitude, the Root Mean Squared Error of Approximation (RMSEA), the comparative fit index (CFI), and the normed fit index (NFI) were used to evaluate the model fit.

**TABLE 2:** The results of Kolmogorov-Smirnov test.

Instrument	Subscales	Kolmogorov-Smirnov Z	Asymp. Sig. (2-tailed)
ER	Situation selection	1.290	0.072
	Situation modification	1.219	0.102
	Attention deployment	1.398	0.064
	Reappraisal	1.104	0.175
	Suppression	1.075	0.198
Self-efficacy	Seeking social support	0.927	0.357
	Efficacy in student engagement	1.106	0.173
	Efficacy in instructional strategies	1.252	0.087
	Efficacy in classroom management	0.908	0.382
L2 grit	Perseverance in teaching	0.698	0.714
	Passion and purpose in teaching	1.364	0.068

Based on Jöreskog (1990), the chi-square is suggested to be non-significant and the chi-square/df ratio should be lower than 3. Moreover, the root mean square error of approximation (RMSEA) should be to be lower than 0.1 (Jöreskog, 1990). The NFI with the cut value greater than 0.90, GFI with the cut value greater than 0.90, and CFI with the cut value greater than 0.90 indicates a good fit (Jöreskog, 1990). According to Table 3, the chi-square/df ratio (2.801) and the RMSEA (0.071) were also acceptable. The other three fit indices, GFI (0.923), NFI (0.951), and CFI (0.914), reached the acceptable fit thresholds.

**TABLE 3:** Fit indices (model 1).

Model	Cut value	
*x* ^2^		114.84
df		41
$x^{2}/df$		2.801
RMSEA	**>**0.1	0.071
GFI	0.9 **<**	0.923
NFI	0.9 **<**	0.951
CFI	0.9 **<**	0.914

**FIGURE 2:**
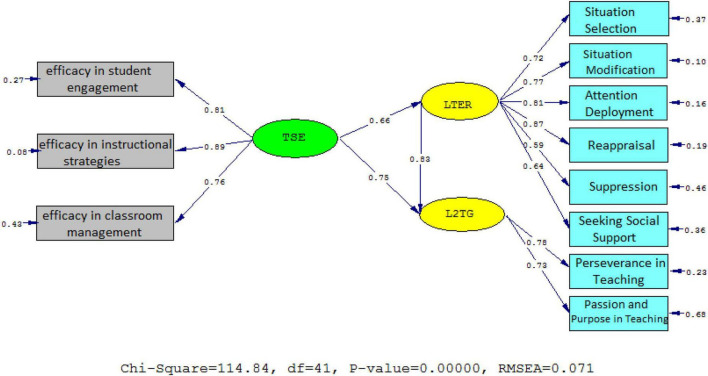
Schematic representation of path coefficient values for the relationships between ER, self-efficacy, and L2 grit (model 1).

**FIGURE 3:**
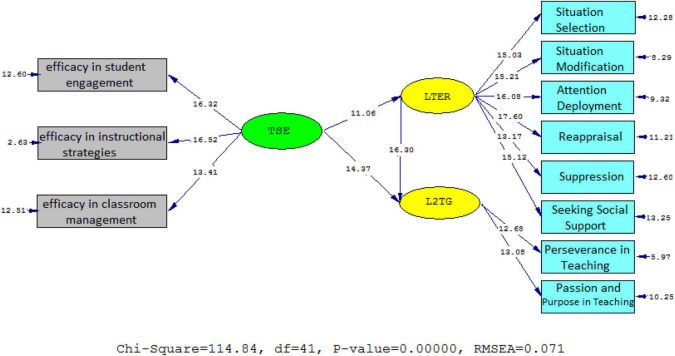
*T*-values for path coefficient significance (model 1).

To check the strengths of the causal relationships among the variables, the *t*-values and standardized estimates were examined. As Figures 2, 3 illustrate, university professor ER affected their sense of efficacy beliefs (β = 0.75, *t* = 14.37) and L2 grit (β = 0.83, *t* = 16.30) significantly and positively; the *t*-value was greater than 1.96. The effect of self-efficacy beliefs on ER was significantly positive (β = 0.66, *t* = 11.06) and the *t*-value was lower than −1.96.

Table 4 presents the acceptable criteria for fit indices in the second model. That is, the chi-square/df ratio (2.884) and the RMSEA (0.073) reached the acceptable fit thresholds. Moreover, GFI (0.934), NFI (0.962), and CFI (0.923) were acceptable.

**TABLE 4:** Fit indices (model 2).

Model	Cut value	
*x* ^2^		648.82
df		225
$x^{2}/df$		2.884
RMSEA	>0.1	0.073
GFI	0.9 <	0.934
NFI	0.9 <	0.962
CFI	0.9 <	0.923

**FIGURE 4:**
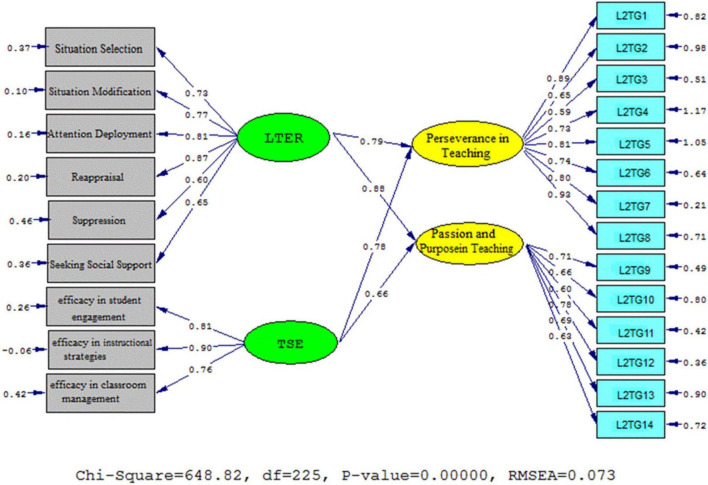
Schematic representation of path coefficient values for the influential role of ER and self-efficacy on L2 grit′ subscales (model 2).

**FIGURE 5:**
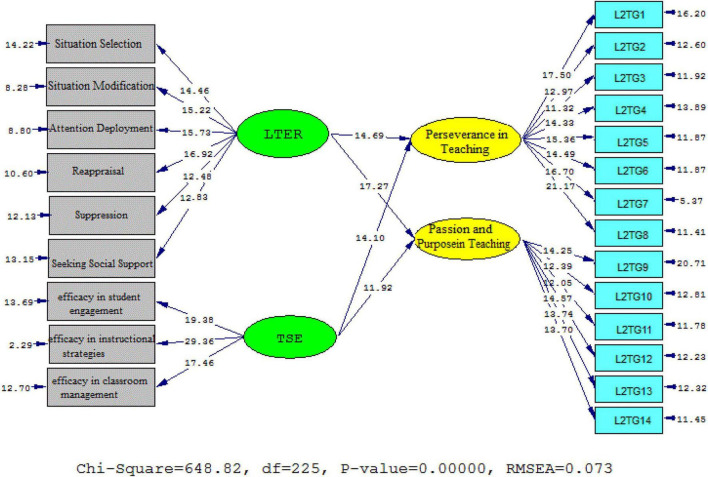
*T*-values for path coefficient significance (model 2).

Figures 4, 5 (Model 2) demonstrate the schematic representation of path coefficient values for the influential role of ER and self-efficacy on L2grit′ subscales. Based on the findings, that university professor ER significantly and positively influenced two sub-components of teacher L2 grit as following: passion and purpose in teaching (β = 0.88, *t* = 17.27) and perseverance in teaching (β = 0.79, *t* = 14.69). The same is true for self-efficacy beliefs and the sub-components of teacher L2 grit. That is, teacher self-efficacy beliefs significantly and positively influenced perseverance in teaching (β = 0.78, *t* = 14.10) as well as passion and purpose in teaching (β = 0.66, *t* = 11.92).

To investigate the relationships between TER, self-efficacy beliefs, and L2 grit, a Pearson product-moment correlation was utilized.

As Table 5 indicates, there were significant relationships among ER and the subcategories of L2 grit were as follows: passion and purpose in teaching (*r* = 0.912, *p* < 0.01) as well as perseverance in teaching (*r* = 0.834, *p* < 0.01). Considering the correlations among self-efficacy beliefs and the sub-components of L2 grit, the results were as following: perseverance in teaching (*r* = 0.834, *p* < 0.01) as well as passion and purpose in teaching (*r* = 0.719, *p* < 0.01).

**TABLE 5:** The correlation coefficients among TSE, LTER, and L2G’ subscales.

	TSE	LTER	Perseverance in teaching	Passion and purpose in teaching
TSE	1			
LTER	0.694**	1		
Perseverance in teaching	0.813**	0.834**	1	
Passion and purpose in teaching	0.719**	0.912**	0.532**	1

**Correlation is significant at the 0.01 level (2-tailed).

## Discussion

The aim of the current study was to uncover the interrelatedness of language TER, self-efficacy, and L2 grit. This aim was accomplished by utilizing a structural equation modeling approach targeting at building a causal structural model by which the contribution of each of the aforementioned constructs can be estimated. Data analyses indicated that ER and self-efficacy skills predict grit tendencies among EFL university professors (see Model 1). The contribution of self-efficacy beliefs to ER was also found (see Model 1). Additionally, the influence of ER and self-efficacy beliefs on the two subcomponents of L2T grit was confirmed (see Model 2). Henceforth, the first null hypothesis (H01. EFL university professors’ ER does not predict their L2 grit.) and second null hypothesis (H02. EFL university professors’ self-efficacy does not predict their L2 grit.) were rejected, and it can be inferred that these constructs (teacher emotion regulation, self-efficacy, and L2 grit) are inextricably interwoven.

### Discussing the first research question

As the data screening suggests, the effect of ER on L2 teacher grit was significantly positive (see Model 1 and 2). It means that ER influenced passion and purpose in teaching (the first subcomponent) and perseverance in teaching (the second subcomponent). It is implied that ER affects university professors’ attitudes and teaching engagement, that leads to their flourishing cognitive accomplishment of them. In other words, teacher ER acts as a campus and gives direction to perform effectively. Scrutinizing the relevant literature on TER and L2 grit presented no identical studies. A recent study in the domain of language learning (Shafiee Rad and Jafarpour, 2022) confirmed that positive emotions interventions influence learner L2 grit, ER, and resilience. Similar findings reflecting the reciprocal relationship between L2 grit, emotions and academic achievement were supported by the results of Ghanbari and Abdolrezapour (2021) as well as Khajavy and Aghaee (2022).

Based on the language TER model (Heydarnejad et al., 2021b), ER is the experience of appraisal, attention deployment, situation modification, seeking social support, situation selection, and to some extent, suppression. According to the teacher L2 grit model (Sudina et al., 2021), teacher L2 grit is assumed as the triggering element provoking perseverance of effort and consistency of interest. Regulation of emotions, which are inevitable parts of teaching improve the strategies that teachers apply (Heydarnejad et al., 2017; teacher’ work life balance and their relationships with students Mulyani et al., 2021). Thus, it can be implied that when university teachers achieve a balance in their emotional states, they can effectively manage their attitudes and skills, which results in responsible decisions. In this regard, Li (2020) noted that grittier teachers are positively engaged and interested in their activities; they make efforts and try to do their best even they face challenges and failures.

### Discussing the second research question

The outcome of the present study also confirmed the predictive power of university professors’ self-efficacy beliefs on their L2 grit (see Model 1). That is, university professors’ beliefs in their ability to effectively handle the tasks, obligations, and challenges related to their professional activity (self-efficacy beliefs) foster dedication to and passion for the teaching (L2 grit). Moreover, the results suggested that university professors’ self-efficacy beliefs correlate positively with teacher L2 grit sub-components. In other words, it is found out that self-efficacy beliefs were associated firstly with perseverance in teaching, and then with passion and purpose in teaching. This result is in accord with the findings of Joy et al. (2020), highlighting the role of self-efficacy skills in boosting grit among school teachers. The intertwined relationships between self-efficacy and L2 grit in the learning context were concluded in the L2 context (Yang et al., 2022). They also emphasize the impact of teachers’ role in helping learners manage their emotions to enhance efficacy and grit. This finding is in line with the outcomes of Shabani et al. (2022). They concluded that teacher L2 grit is the product of self-reflection, efficacy skills, and affective evaluation.

Bandura’s self-efficacy theory (Bandura, 1982), as the foundation of teacher self-efficacy theory, demonstrates that efficacious teachers implement mastery experiences for cognitive development, which is a powerful predictor of teaching performance. Self-efficacy has become an important framework in education to predict and explain the perceptions and judgments that influence teachers’ and university professors’ decisions and actions in the classroom. The Grit theory (Duckworth, 2016) as well as teacher L2 grit (Sudina et al., 2021), suggest that perseverance and passion for overcoming challenges are inevitable parts of gritty teachers’ traits. This personality trait helps them expand their efforts and keep enthusiastic despite obstacles and inadequate progress. Thus, the results of this study can be interpreted through the lens of these theories that affirm self-efficacy serves to manage teachers’ cognitions and emotions, leading to higher and longer professional development (Duckworth and Gross, 2014).

Additionally, ER was discovered to be significantly affected by self-efficacy beliefs as well (see Model 2). According to these data, it can be inferred that quite apart from its direct influences, self-efficacy appeared to assist university professors in modulating their experienced emotions. That is to say, the levels of teacher efficacy positively correlate with increased ER among EFL university professors. This result corroborates with those of Heydarnejad et al. (2021a) and Xiyun et al. (2022), as well as Fathi et al. (2020). Moreover, the findings from Zee and Koomen (2016) and Cansoy et al. (2020) evinced that teacher self-efficacy and their psychological wellbeing are interwoven. Based on self-efficacy theory (Bandura, 1982), teacher efficacy develops from a combination of mastery experience, vicarious experience, social persuasion, and physiological and emotional states. It can be inferred that all of them are directly and indirectly connected to components of the language TER model (i.e., situation selection, situation modification, attention deployment, reappraisal, suppression, and seeking social support). This means that efficacious university professors have high levels of self-awareness, self-worth, and self-perception; thus, they can positively guide actions and responses to the emotional demands of their teaching. Consequently, they can develop relatively positive emotion regulatory strategies that pave the way for stability in the teaching profession.

## Conclusion

This investigation highlighted the significant contribution of ER and self-efficacy to L2 grit, and provided strong empirical confirmation that via the help of ER and self-efficacy, university professors can ameliorate their practice for a longer period of time even in the face of teaching chaos and complexities. This leads to a positive attitude toward the teaching profession in higher education, which heightens success instead of failure. Furthermore, the present study reflected that the effect of university professors’ self-efficacy on ER is also significant. Taken together, these findings suggest the predictive role of ER and self-efficacy in promoting L2 grit. In the domain of language teaching, especially in higher education, exploring the relationship between these constructs is quite rare. Thus, this domain is a fertile field and calls for more empirical studies, which pave the path for promoting teachers’ wellbeing and effective pedagogy.

### Implications

The findings of this study suggest some pedagogical implications for educators in higher education. The knowledge about situational and personality determinants of ER strategies and their efficiency is vital and should be considered in higher education program. Such training programs should concentrate on practicing the broad repertoire of strategies and showing the conditions under which, they are effective or not. Moreover, training should focus on reflecting more on university professors’ own traits and preferences that may influence the effectiveness of their employed ER strategies. This information, also incites university professors to alter or modify their employed ER strategies to more positive ones, which are in turn expected to facilitate their self-efficacy beliefs and L2 grit.

In the area of language teaching training programs, some international centers such as TESOL and CELTA are preparing specialized language teachers around the world. In Iran, pre-service and in-service teacher training programs are held, and teacher trainers can make a significant contribution by instructing EFL university professors about the importance of emotions and ways to regulate and modify their emotions. Furthermore, the implications of this research would be of great help to be considered in pre-service and in-service teacher training programs, which are usually held for university professors. Practicing self-aid skills (i.e., self-evaluation, self-efficacy, self-awareness, and self-regulation) in pre-service and in-service teacher training pogroms, particularly in higher education could boost L2 grit and foster efficient teaching, particularly during the Covid-19 pandemic and university lockdown. These programs are expected to pinpoint the effective path for enhancing their effective teaching.

### Limitations and suggestions for future researchers

The findings of this research suffer from some limitations: Firstly, this study employed quantitative design. To have a deeper understanding of the causal links among the variables, future research can apply mixed-method approaches to delve into the relationship between university professors’ ER, self-efficacy, L2 grit, and other teacher-related constructs (e.g., work engagement, autonomy, critical thinking, job satisfaction, reflective teaching, self-regulation, and immunity). Secondly, the effects of participants’ demographic variables on ER, self-efficacy beliefs, and L2 grit were not explored, which can be a suggestion for future research. Lastly, due to practical constraints, the participants were chosen according to convenience sampling. Which is not truly representative. Therefore, results from this study should be interpreted and generalized with great caution. Concerning delimitations, this study is to focus on the employed ER strategies by EFL university professors at the workplace. Furthermore, to assess EFL university professors’ ER, a trait approach was utilized. That is, frequently and intensively ER strategies in response to emotional experiences at the workplace were assessed retrospectively.

As a future research avenue, it is recommended to use a new method of data gathering called the Experiential Sampling Method (ESM) in future studies, which can assess emotions at the intra-individual level. The experience sampling method (ESM) is used to evaluate emotions contextualized in real-world settings. This method helps researchers decrease memory biases and increase ecological validity, and hypothesize testing at the between- and within-person levels. Furthermore, it is recommended to undertake further research to explore whether TER influences their learners’ ER. As a further suggestion, researchers can explore the relationships between ER, self-efficacy, and L2 grit in other educational contexts such as schools and private language institutes.

## Data availability statement

The original contributions presented in this study are included in the article/supplementary material, further inquiries can be directed to the corresponding author.

## Author contributions

All authors listed have made a substantial, direct, and intellectual contribution to the work, and approved it for publication.
